# Avicenna's Canon of Medicine: a review of analgesics and anti-inflammatory substances

**Published:** 2015

**Authors:** Shahla Mahdizadeh, Maryam Khaleghi Ghadiri, Ali Gorji

**Affiliations:** 1 *Epilepsy Research Center, Universitätsklinikum Münster, Münster, Germany*; 2 *Department of Neurosurgery, Universitätsklinikum Münster, Münster, Germany*; 3 *Shefa Neuroscience Research Center, Tehran, Iran*; 4 *Department of Neurology, Universitätsklinikum Münster, Münster, Germany*

**Keywords:** *Medicinal plants*, *Medieval period*, *Pharmacology*, *Pharmacotherapy*, *Phytotherapy*

## Abstract

Naturally occurring substances mentioned in medieval medical literatures currently have, and will continue to have, a crucial place in drug discovery. Avicenna was a Persian physician who is known as the most influential medical writers in the Middle ages. Avicenna`s Canon of Medicine, the most famous books in the history of medicine, presents a clear and organized summary of all the medical knowledge of the time, including a long list of drugs. Several hundred substances and receipts from different sources are mentioned for treatment of different illnesses in this book. The aim of the present study was to provide a descriptive review of all anti-inflammatory and analgesic drugs presented in this comprehensive encyclopedia of medicine. Data for this review were provided by searches of different sections of this book. Long lists of anti-inflammatory and analgesic substances used in the treatment of various diseases are provided. The efficacy of some of these drugs, such as opium, willow oil, curcuma, and garlic, was investigated by modern medicine; pointed to their potent anti-inflammatory and analgesic properties. This review will help further research into the clinical benefits of new drugs for treatment of inflammatory diseases and pain.

## Introduction

Abu Ali al-Ḥusain Ebn Abdullah Ebn Sina (980 –1037 AD), known in the West as Avicenna, was a Persian physician who is known as the most influential medical writers in the medieval period. Between the thirteenth to the seventeenth centuries, Avicenna held a high place in Western medical studies, ranking as an acknowledged supremacy. His works had a crucial impact on the modern medicine and at some universities continued to be used for teaching up to the nineteenth century (Brentjes, 1980 ▶; Weisser, 2011 ▶).

About 100 dissertations were written by Avicenna. Among them, *Qanoon-fel-teb* (The Canon), originally written in the Arabic, is an immeasurable encyclopedia of medicine that represents all the medical sciences of the time. The Canon was translated into several languages, including Latin, Persian, English, Indian, Chinese, Hebrew, German, and French (Hakim Syed Zillur Rahman, 2004 ▶; Weisser, 2011 ▶). The Canon, translated first into Latin (*Canon medicinae*) by Gerard of Cremona, was the main medical textbook in several universities in Europe between 11^th^ to 17^th^ centuries (Moosavi, 2009 ▶). Sir William Osler, one of the four founding professors of Johns Hopkins Hospital, noted the Canon as "the most famous medical textbook ever written; a medical bible for a longer time than any other work" (Osler, 1972 ▶).

Avicenna (1988) ▶ divided the Canon into five books:

1. The first book describes different scope of medicine, the elements, the temperaments, the humors, physiological and anatomical principles, and general therapeutic procedures. 

2. The second book explains several plant-, animal-, and mineral-derived drugs, in alphabetical order, with an essay on their properties and side-effects. 

3. The third book describes an organ-based classification of the diagnosis and treatment of different diseases.

4. The fourth book defines general medical problems that affect the whole body, such as obesity, poisonous bites, and fever.

5. The fifth book contains numerous compound receipts.

Eight hundred drugs and 650 recipes of different compounds from various sources are listed in the Canon, with extensive comments on the effectiveness of each drug or recipe (Danielle, 2008 ▶). Several clinical and experimental studies support the use of some traditional Persian medicines noted by Avicenna in the Canon for the treatment of different diseases. However, most medicinal substances prescribed by Avicenna remain largely unexamined (Gorji, 2003 ▶). The anti-inflammatory and analgesic medicaments are a heterogeneous group of substances, which share definite remedial actions and side effects. The search for new pharmacologically active anti-inflammatory and analgesic drugs offered by medieval medical literatures has led to the discovery of some clinically useful drugs. These compounds, during the last two centuries, have played a crucial role as effective remedies of various human diseases as well as in understanding of basic pathophysiology of some diseases (Gorji and Khaleghi Ghadiri, 2001 ▶; Gorji and Khaleghi Ghadiri, 2002 ▶; Khaleghi Ghadiri and Gorji, 2004 ▶; Vakili and Gorji, 2007 ▶; Bayan et al., 2013 ▶). Despite progress in the development of therapy in recent years, effective and potent anti-inflammatory and analgesic drugs are still required for the treatment of different diseases. The aim of the present study was to provide a descriptive review of all anti-inflammatory and analgesic drugs noted in the Canon of Avicenna. Data for this review were provided by searches of different sections of this book translated in Persian (Avicenna, 1988 ▶). It is hoped that this manuscript will help further research into the clinical benefits of these compounds for treatment of inflammatory diseases.

**Anti-inflammatory and analgesic drugs**

Avicenna (1988) ▶ describes the signs and symptoms (pain, swelling, redness, fever, impaired functions, etc.) of different inflammatory diseases (such as pneumonia, rhinitis, otitis, dermatitis, etc.) and their treatment with several substances with different modes of action. These drugs were advised to use as prophylactic or therapeutic. Many of these drugs were prescribed for different inflammatory disease, although some of those were advised for a certain disorder. The strategies for treatment of pain and inflammation were divided into the measures of acute and chronic diseases. 

Furthermore, these drugs were classified for treatment of mild, moderate, or severe conditions in each disorder. It is also noted that some of these drugs in certain conditions act as anti-inflammatory of analgesic drugs, in other condition my provoke pain or inflammation. The anti-inflammatory and analgesic drugs listed in the Canon include plants, animal products, and minerals. Avicenna emphasized the importance of the dose and the route of administration and defined a schedule for drug application. Drugs were also taken via skin, oral, nasal, or rectal routes as well as by inhalation (Table).

Some of the medicaments suggested by Avicenna for treatment of inflammation and pain are well-recognized dugs in modern medicine. Many of these compounds are under experimental or clinical investigations for their probable therapeutic effects. However, most of these drugs remain largely unexamined.

**Table:** Anti-inflammatory and analgesic substances used in medieval Persia by Avicenna and noted in The Canon. AI (Anti-inflammatory); AG (Analgesic).

***Latin Name***	**Common Name **	**Effect**	**Administratin**	**Diseases**
*Acorus calamus*	Sweet flag	AG AI	Oral Locally	Sciatica Headache Toothache Pneumopleuritis Mastitis Fever
*Acorus calamus*	Sweet flag	AG	Oral Locally	Muscle pain Sciatica Colic Uterine pain Fever Pneumonia
*Adiantum capillus-veneris*	Maidenhair	AI	Locally	General edema
*Allium ascalonicum *	Shallot	AG AI	Oral Locally	Headache Arthritis Otitis
*Allium sativum*	Garlic	AG AI	Oral Locally	Acute Inflammation Chronic and Malignant Wounds Arthritis and Gout Sciatica Common cold Headache Earache Severe Eye Pain Acute Cough Lung disease with hematemesis Gastroenteritis Liver diseases
Almond oleum	Almond oil	AG AI	Locally	Rhinitis
*Alsidium Helminthocorton*	Corsican	AG	Oral Locally	Intestinal ulcer Uterine and cervical pain
*Althaea officinalis*	Marshmallow	AG AI	Locally Oral	Dermatitis Arthralgia Sciatica Earache Toothache Chronic fever
*Anacyclus pyrethrum*	Pellitory	AG	Oral Locally	Dermatitis Tongue swelling Headache Encephalitis Earache Toothache
*Anemon cronaria*	Anemone	AG	Oral Locally	Arthritis Neuralgia
*Pimpinella anisum*	Aniseed	AG AI	Oral Locally	Enteritis Orchitis Sores in nose Tooth pain
*Anethum graveolens*	Dill	AG AI	Oral Locally	Chronic skin wounds Arthritis and Gout
*Apium graveolens*	Wild celery	AG AI	Locally Oral	Orchitis Inflammation of the oral Cavity Headache
*Aquilaria malaccensis*	Aloes	AG AI	Oral Locally	Toothache
*Artemisia absinthium*	wormwood	AG AI	Oral Locally Inhalation	Otitis Chronic ophthalmitis Chronic fever
*Artopa belladona*	Nightshade	AG	Locally	General pain Gastroenteritis
*Asarum europaeum*	Cabaret	AG AI	Oral Locally	Sciatica Chronic ophthalmitis Fever
*Asparagus officinalis*	Asparagus	AG AI	Oral Locally	Abdominal pain
*Asphodelus ramosus*	Asphodel	AG AI	Locally	Dermatitis Purulent otitis Gastritis
Avenae* fatua*	Oat	AI	Oral Locally	Scalp inflammation Eczema Earache
*Bambagia*	Cotton plant	AG AI	Oral Locally	Earache Sore Throat Cough Chest pain Toothache
*Berberis vulgaris*	Barberry	AI AG	Locally	Arthritis
*Boswellia serrata*	Frankincense	AI AG	Oral Locally	Scalp inflammation Arthritis Gout
*Brassica oleracea*	Cabbage	AI AG	Oral Locally	Dermatitis such as herpes Toothache Cough Bronchitis and shortness of Breath Chest infections Eye pain Dropsy and edema Anal fissure
*Brassica Spp.*	Mustard	AG	Locally	Joint and muscle pain
*Cannabis Sativa*	Hemp	AI	Locally	Ophthalmitis General edema Infectious wounds Gout Uterine pain
*Carthamus Tinctorius*	Safflower	AG	Oral Locally	Muscle pain Headache Toothache and dental caries Chronic earache Chronic cough and bronchitis Purulent wounds
*Carum carvi*	Caraway	AI AG	Oral Locally	Edema Arthralgia Muscle discomfort Arthralgia Scalp inflammation
*Cheese*	-	AG	Oral Locally	Pleuritis Gastrointestinal discomfort
*Cassia fistula*	Golden shower	AI AG	Oral Locally	General edema Earache Scalp inflammation Joint and bone pain Eye wounds Gastritis
Castor oleum	Castor oil	AI	Oral Locally	Scalp inflammation Chronic headache
*Centaurea cyanus*	Cornflo-wer	AI AG	Oral	Colic
*Chrysanthemum parthenium*	Feverfew	AG	Locally	Sciatica
*Cicer arietinum*	Chana	AI AG	Locally	Eye swelling Hoarseness Bronchitis and cough Stomach pain and gastric ulcer Uterine pain and infection Intestinal ulcer Gastroenteritis Colic
Cichorium intybus	Chicory	AI AG	Oral Locally	Gum disease Gastritis Headaches Earache Eye swelling Intestinal ulcer
*Cicuta maculata*	Hemlock	AI AG	Oral Locally	Malignant and purulent Wounds Rhinitis Chronic headache Toothache Ophthalmitis
*Cocos nucifera*	Coconut	AI AG	Oral Locally	Neck pain Gastrointestinal ulcer Uterine and gum discomfort
*Colchicum autumnale *	Meadow saffron	AI AG	Oral Locally	Purulent dermatitis Burn Chronic wounds Sciatica Arthralgia Toothache Headache
*Commiphera myrrha*	Myrrh	AI AG	Locally	Arthralgia
*Commiphora gileadensis*	Balsam of mecca	AI AG	Oral Locally Inhalation	Pleuritis Headache Gastritis Bronchitis Kidney pain Hemorrhoid
*Convolvulus scammonia*	scammony	AI AG	Oral	Gastroenteritis
*Coriandrum sativum*	Coriander	AI AG	Oral Locally	Muscle weakness Artralgia Headache Purulent otitis Bleeding gums and gingivitis Gastroenteritis Hemorrhoid
*Corylus aveilana*	Common hazel	AI AG	Oral Locally	General edema Herpes Accidental injuries Muscle painHiccups Gastritis Chronic fever
*Crocus sativus*	Saffron	AI AG	Oral Locally	Acute edema and abscess Bone fractures Joint and tendon pain Headache Oral diseases Diphtheria
*Cucurbita pepo*	Pumpkin	AI AG	Oral	Genitourinary pain
*Cupressus sempervirens*	pencil pine	AI AG	Locally	Chronic wounds Arthralgia Gout
*Curcuma longa*	Curcuma	AI AG	Oral Locally	Dermatitis Pain in the mouth and gum Scalp wounds
*Cyclamen coum*	Sowbread	AG	Locally	Chronic toothache
*Cymbopagon schoenantus*	Sweet rush	AI AG	Oral Locally	Muscle pain Metritis Cervicitis
*Cynara cardunculus *	Artichoke	AI AG	Oral Locally Inhalation	Headache Colic Sciatica Arthralgia Bronchitis
*Dausus carota*	Carrot	AG	Oral Locally	Kidney and uterine pain Fever
*Elettaria cardamomum*	Cardamom	AI AG	Oral Locally	Sciatica
*Chees Ferment*	-	AI AG	Oral Locally	Otitis Cystitis Gastritis Colic Fissure anal
*Faba vulgaris*	Broad bean	AG	Oral Locally	Diphtheria Ophthalmitis Otitis Abscess Gastrointestinal pain Arthritis
Ferula assafoetida	Stinking gum	AI	Oral	Fever
*Ferula gumosa*	Galbanum	AI AG	Oral Locally	Headache Gastroenteritis
*Ficus carica*	Comm-on fig tree	AI AG	Oral	Malignant and purulent Wounds Stomatitis Tooth Pain Gastroenteritis
*Foeniculum vulgare*	Fennel	AI AG	Oral	Stomatitis Otitis Gastritis
*Flores acacia*	Acacia	AI AG	Oral Locally Inhalation	Arthritis Chronic eye disease
*Fraxinus excelsior*	Ash tree	AI AG	Locally	Gout Arthritis Earache
*Fumaria perriflora*	Fineleaf fumitory	AG	Locally	Back pain Toothache Gingivitis
*Gentiana lutea*	Gentian	AI	Locally	Abdominal pain
*Glycyrrhiza glabra*	Liquorice	AI AG	Oral Locally	Skin wounds Scabies Arthralgia Back pain Chronic headache
*Hedera helix *	Ivy	AI AG	Oral Locally	Skin infections Earache Scalp inflammation
*Helleborus niger*	Christmas rose	AI AG	Locally	Tonsillitis Arthritis Gout Liver pain
*Hordeum vulgare*	Barley	AI	Oral	Fever
*Hyssopus officinalis*	Hysso	AG	Locally	Eye swelling Otitis Uterine diseases
*Illicium verum *	Anise	AI AG	Oral Locally Inhalation	Dizziness Otitis Headache Chronic ophthalmitis Chronic fever
*Iris florentina *	Iris	AI AG	Oral Locally	Toothache Joint contortion
*Jasminum officinale*	White jasmin	AI AG	Locally	Painful skin disease Acute mastitis (in pregnancy) Earache Ophthalmitis Eye tumor Uterine pain Gastroenteritis Painful sores in the anal area
*Juglans regia*	Walnut	AG	Oral	Cervical pain General pain
*Lactuca sativa*	Lettuce	AI AG	Oral Locally	General edema Joint complaints and gout Sciatica Stomatitis Uterine pain
*Lantago psyllium*	Flea wort	AI	Locally	Gingivitis
Laureo oleum	Laurel oil	AI	Locally	Joint diseases and gout Ophthalmitis Neck pain Sore throat
*Lavendula stoechas*	Spanish lavender	AI AG	Locally	Arthritis Anal fissure
*Lawsonia intermis*	Henna	AI AG	Oral Locally	Abscess Purulent scalp inflammation Purulent rashes Sciatica Bronchitis
*Leidium sativum*	Garden cress	AI AG	Oral Locally	Gastrointestinal infections Osteitis Purulent otitis Toothache Intestinal ulcer Hemorrhoids
*Lens culinaris*	Lentil	AI	Oral	Intestinal pain
Lignum vite	Grape Tree	AI AG	Oral Locally	Earache Headache
*Lilium candidum*	Iris	AI AG	Oral Locally	Eye infections Sore throat Pyelonephritis Cystitis Chronic fever
*Malus orientalis*	Apple	AI AG	Oral Locally	Acute general edema Muscle pain Abscess Otitis General pain Malignant purulent wounds Toothache Chronic cough and pneumonia Abdominal pain Gastroenteritis
*Marrubium vulgare*	Marrubium	AG	Oral Locally	Sciatica Arthritis Gout Otitis Mouth wound Gastroenteritis Colic
*Matricaria Spp*	Camomile	AI AG	Oral Locally	Toothache Muscle tightness
*Meliloto officinalis*	Field melilot	AI AG	Oral Locally	Otitis Headache Eye swelling
*Mentha piperata*	Pepper mint	AI AG	Locally Oral	Purulent skin wounds Pneumonia Mastitis
*Mentha pulegium*	Penny royal	AI AG	Oral Locally	Arthralgia Purulent Malignant sore
*Morus alba*	White mulberry	AI AG	Oral Locally	Abscesses and rash Herpes Purulent dermattis Sciatica Malignant sore Toothache Sore throat Chest pain Chronic cough
*Myristica fragrans*	Nutmeg	AI	Oral Locally	Arthritis Epilepsy Gout Headache Toothache Cystitis Gastrointestinal pain Cerebrale Kidney pain Arthralgia
*M* *yrtus communis*	Myrtle	AI AG	Oral Locally Inhalation	Testitis Headache Arthritis Otitis Chronic eye disease Gingivitis Cystitis Urinary tract infection Hemorrhoid
*Narcissus pseudonarcissus*	Lent lily	AI AG	Oral Locally	Headache Hardness tongue Earache Mastitis
*Nerium oleander*	Rosebay	AI	Oral Locally	Acute inflammatory Cervical pain
*Nigella sativa*	Black cumin	AI AG	Oral Locally	Arthralgia Earache Abdominal pain Hemorrhoids Anal fissures Sores in the genital area
*Ocimum basilicum*	Basil	AI AG	Oral Locally	Kidney-bladder pain Abscess Headache Toothache Gastritis Hepatitis Menstrual pain Uterine pain Arthralgia Intestinal ulcer Ophthalmitis
*Olea europaea*	Olive	AI AG	Locally	Gout Acute ophthalmitis Chronic swelling of the diaphragm Liver disease Uterine pain Kidney pain
Oleum lilia	Lilies oil	AI	Locally	Earache Headache Tinnitus Kidney pain Bladder pain Uterine discomfort
*Onosma echioides*	Hairy onosam	AG	Oral Locally	Stomachache Lumbar pain Liver pain
Opoponax gummi	Opopana-ax Gum	AG	Locally	Pain Sciatic pain
*Orchis maculata*	Spotted orchis	AG	Oral Locally	Skin diseases Herpes Skin induration Corns Arthritis Tinnitus and hearing loss Headaches Eye diseases Cystitis Gastroenteritis
*Origanum majorana*	Marjoram	AI AG	Oral Locally	Dermatitis Headaches Ophthalmitis Lung diseases such as bronchitis and chronic cough Uterine pain Chronic fever Toothache Gingivitis Otitis
*Origanum vulgare*	Wild marjoram	AI AG	Oral Locally	Toothache Swelling of the spleen
*Oxalis crenata*	Sorrel	AI AG	Oral Locally	Arthralgia Purulent lung diseases Blood spitting and coughing
*Paeonia officinalis*	Common peony	AI AG	Oral Locally	Toothache Flatulence Abdominal pain and fever
*Papaver somniferum*	Opium poppy	AI AG	Oral Locally	Arthralgia Sciatica Gout Muscle pain and nerve injury Otitis Blepharitis Urogenital pain Abdominal pain Uterine pain Colic Postoperative pain Chronic pain
*Papavero rosolaccio*	Red poppy	AI AG	Locally	Dermatitis Earache Eye pain Uterine pain Orchitis
*Parce carduus*	Thistle	AI AG	Oral	Chronic uterine/cervical pain
*Pastinaca sativa*	Pastinace	AI AG	Oral Locally	Headache Stomatitis Ophthalmitis Dermatitis Fever
*Phonix dactylifera*	Date	AI AG	Locally	Ophthalmitis Toothache
Pinus grana	Pine seeds	AI AG	Oral Locally	Abscess Dental caries Toothache Spleen diseases Uterine disorders
Pinus nuces	Pine cone	AG	Locally	Back muscles vertebrae pain
*Pinus silvestris*	Pine	AG	Oral	Arthralgia Nerve pain Back pain Muscle complaints Abdominal pain and colic
*Pinus sylvestris*	Scots pine	AI AG	Oral Locally	Pleuriti Hepatitis Splenitis Gastroenteric complain Joint and bone pain Ophthalmitis
*Piper nigrum*	Black pepper	AI AG	Oral Locally	Chronic earache
*Pistacia vera*	Pistachio	AG	Oral Locally	Dermatitis Eliminates the fever Headache
*Plantago major*	Way-bread	AI AG	Locally	Bone fracture Gout Arthralgia
*Platanus orientale*	Oriental plane	AI AG	Locally	Knee pain Back pain Toothache General hard edema
*Polygonum amphibium*	Water knotweed	AI AG	Locally	Lumbar pain
*Polypodium vulgare*	Adders-fern	AI AG	Oral Locally	Tonsillitis Otitis Arthritis Ophthalmitis Mastitis Chronic cough
Populiferve	Poplar	AI AG	Oral Locally	Headache
*Portulaca oleracea*	Common purslane	AI AG	Oral Locally	Headache Arthritis Testitis
*Pruntus domestica*	Plum	AI AG	Oral	Spleen pain General inflammation
*Pterocarpus santalinus*	Sandal wood	AI AG	Locally	Tooth pain Chronic cough Liver pain
*Punica granatum*	Pomegranate	AG	Oral	Gastroenteritis (particularly in children) Cervical discomfort Arthralgia Back pain Headaches Earache Colic Dermatitis Ophthalmitis Ocular diseases
*Raphanus sativus*	Radish	AI AG	Oral	Hepatitis
*Recinus*	Castrol oil	AI	Locally	General edema Purulent skin wounds Impetigo
*Rhabarber rhaponticum*	Rheum	AI AG	Oral Locally	Gastritis Gonorrhea Chronic fever
*Rhus coriaria*	Sicilian sumac	AI AG	Oral	Gastritis Spleen pain Metritis Kidney pain
*Rosa gallica*	Red rose	AI AG	Oral	Edema Toothache Earache Breast disease Bronchitis Cough
Rosam	Rose oil	AI AG	Locally	Sciatica and back pain Toothache Headache
*Rubus* *sectio *	Bramble	AI	Oral Locally	Arthritis Gout Acute ophthalmitis Mastitis Colitis
*Saccharum officinalis*	Sugar cane	AI AG	Oral Locally	Gastritis Hepatitis Metritis
*Salix*	Willow	AI AG	Oral Locally	Osteitis Headache Ophthalmitis Orbital Injury Liver disorders and jaundice Dysmenorrhea
*Seasam indicum*	Sesame	AG	Locally	Chronic ocular disease Earache Hemorrhoid
*Semecarpod oriente*	Marsh nut	AI AG	Locally	Colic Arthralgia
*Sesamum indicum*	Sesame	AI AG	Oral Locally	Chronic and malignant sore Burn Toothache Earache Mastitis
*Styrax officinalis*	Styrax tree	AI AG	Oral Locally	Malignant tumors Acute earache Toothache Rash Ophthalmitis Bronchitis Kidney and bladder pain Intestinal ulcer Hemorrhoids Fever
*Sulfur*	Sulfur	AI AG	Oral Locally	Orchitis Ophthalmitis Colic
*Tamarindus indica*	Tamarind	AI	Oral Locally	Dermatitis Acute wounds stomach pain Gastroenteritis
*Tamarix gallica*	Tamarisk	AG	Locally	Acute edema Headaches with fever Stomatitis
*Taraxacum officinale*	Dandelion	AI AG	Locally	Abscess Arthralgia Deep wound Bone wound Dermatitis Headache Purulent ear infections Injury of the orbits
*Taxus baccate*	Yew tree	AI	Oral Locally	Sciatic pain Arthritis and gout Dental pain and dental caries Headaches Ear Diseases Gum bleeding and gingivitis
*Tragopogon pratensis*	Meadow salsify	AI AG	Locally	Wounds and nerve damage Muscleaches Gastroenteritis
*Trigonella foenum-graecum*	Alhova	AI AG	Oral Locally	Sciatic pain Arthralgia
*Tropaeolum majus*	Monks cress	AI AG	Oral Locally	Sore throat Arthralgia
*Urtica dioica*	Nettle	AI AG	Oral Locally	Gout Headache Otitis Ophthalmitis Diarrhea Colic Chronic pain
*Valeriana officinalis*	Valerian	AI AG	Oral Locally	Headache Inflammation of the penis Ophthalmitis
*Verbascum thapsiforme*	Mullein	AI AG	Locally	Rhinitis Sinusitis Otitis Orchitis Ophthalmitis
*Vicia sativa*	Tare	AG	Locally	Burn Arthralgia Headache Uterine pain
*Vinegar*	-	AG AI	Oral Locally	Herpes Purulent skin wounds Gout Headache Gingivitis
*Viola odorata*	Sweet violet	AI AG	Locally	Headache Cold
*Vitex agnus-castus*	Chasteberry	AG	Oral Locally	Pain Colic
*Vitis vinifera*	Grape vine	AG	Oral Locally	Gasteritis Uterine pain Abdominal pain Kidney and bladder pain
*Zingiber officinale*	Ginger	AI AG	Oral Locally	Headache General pain

**Well recognized anti-inflammatory and analgesic drugs**

*Papaver somniferum*

Opium (*Papaver somniferum*) was advised for treatment of arthralgia, sciatica, gout, muscle pain and nerve injury, otitis, blepharitis, urogenital pain, abdominal pain, uterine pain, colic, postoperative pain, and chronic pain in the Canon. In 1680, Sydenham was noted opium: "Among the remedies which it has pleased Almighty God to give to man to relieve his sufferings, none is so universal and so efficacious as opium" (Yaksh and Wallace, 2011 ▶).

 Administration of opiate in Europe increased rapidly in the 18th century (Miller and Tran, 2000 ▶). Opium and its derivatives have been used as the most widely analgesics for severe pain since the early 1800s (Hamilton and Baskett, 2000 ▶). Nowadays, application of several opioids is considered effective for the treatment of various forms of headaches (Gorji and Khaleghi Ghadiri 2001 ▶), postoperative pain (Hamilton and Baskett, 2000 ▶), neuropathic pain (Berrios et al., 2008 ▶), and different chronic pain syndromes (Vallejo et al., 2011 ▶). Opium also plays a crucial role in our understanding of basic mechanism of pain (Lipman, 1990 ▶).

*Salix spp.*

In the Canon, administration of willow oil (*Salix spp.*) was recommended for treatment of headache, osteitis, ophthalmitis, orbital injury, liver disorders and jaundice, and dysmenorrhea. In the first half of the 19th century salicin, the principal active constituent of willow oil was extracted from the willow bark and later salicylic acid was obtained. Today, the synthetically produced preparations of salicylic acid are well-known analgesic, anti-inflammatory and antipyretic drug (Amann and Peskar, 2002 ▶). 

Acetylsalicylic acid is recommended as an analgesic and prophylactic in different types of headaches [4], and alleviates dysmenorrhea (Pendergrass et al., 1985 ▶). Furthermore, it has been suggested that regular aspirin use (more than 15 times per month) may be associated with a lower prevalence of non-alcoholic fatty liver disease among men and older patients (Shen et al., 2014 ▶). 

*Curcuma longa*

Curcuma (*Curcuma longa*) is advised for treatment of different inflammatory diseases and pain in the Canon. Promising effects of curcuma have been reported in patients with various pro-inflammatory diseases, including oncologic disorders, cardiovascular disease, rheumatologic diseases, chronic anterior uveitis, gastrointestinal inflammatory diseases (Crohn's disease, ulcerative proctitis and colitis, irritable bowel disease, pancreatitis, gastric inflammation as well as ulcer, and cholecystitis), lupus nephritis, ischemic brain injuries, and acquired immunodeficiency syndrome (Gupta et al., 2013 ▶; Tamaddonfard, 2013 ▶; Arshami et al., 2013 ▶; Ghosh et al., 2014 ▶). 

*Cannabis sativa*

Cannabis (*Cannabis sativa*) was prescribed for the alleviation of severe headache as well as treatment for degenerative bone and joint diseases, ophthalmitis, general edema, infectious wounds, gout, and uterine pain. The major active component of cannabis, tetrahydrocannabinols, in addition to other constituents of cannabis has been shown to possess anti-nociceptive properties (Wilson and Nicoll, 2002 ▶). 

Cannabinoids alleviate pain by the activation of a brainstem circuit that is required for opioid-mediated analgesia, and modulate basal nociceptive thresholds through the activation of the rostral ventromedial medulla [8]. It has been suggested that cannabinoids may act as an analgesic in migraine pain by inhibition of spreading depression phenomenon (Kazemi et al., 2012 ▶). Different constituents of cannabis have been suggested to be useful in the treatment of intervertebral disc degeneration (Silveira et al., 2014 ▶), endometriosis (Sanchez et al., 2012 ▶), and breast cancer (Behrend, 2013 ▶). Cannabis has been suggested to be used to treat patients with cancer who do not adequately treated with other analgesics and anti-emetics (Nauck et al., 2004 ▶).

*Allium sativum*

Garlic (*Allium sativum*) was recommended by Avicenna in his book for treatment of acute inflammation, chronic and malignant wounds, arthritis and gout, sciatica, common cold, headache, earache, severe eye pain, acute cough, lung disease with hematemesis, gastroenteritis, and liver diseases. Anti-inflammatory effect is a well-known property of this plant. Garlic and its bioactive components protect the hepatocytes from several toxic agents and act as antimicrobial, antifungal, and antiviral substances (Bayan et al., 2014 ▶). Diallyl disulfide, a major organosulfur compound in garlic oil, has been shown to a useful substance in treatment of respiratory inflammation (Shin et al., 2013 ▶). Administration of garlic have been suggested for treatment of common cold (Allan and Arroll, 2014 ▶), arterial occlusive disease (Jepson et al., 2013 ▶), migraine headache (Roussos and Hirsch, 2014 ▶; Marschollek et al., 2014 ▶), and prevention of different tumors (Bayan et al., 2014 ▶). 

**Medicaments under investigation for their probable anti-inflammatory and analgesic effects**

*Matricaria chamomilla*

Avicenna advised to use chamomile (*Matricaria chamomilla*) for treatment of headache, edema, conjunctivitis, jaundice, chronic fever, lithiasis, amenorrhea, toothache, and muscle tightness. Chamomile is recommended to relieve itching and inflammation and facilitate healing of peristomal skin lesions in patients undergone the gastrointestinal or urinary surgeries (Charousaei et al., 2011 ▶).

The fluid extract from chamomile reduced pain of aphthous ulcers in patients suffering from recurrent aphthous stomatitis (Ramos-e-Silva et al., 2006 ▶). It is hypothesized that chamomile flavonoids and polyphenols due to its anti-inflammatory properties via the inhibition of pro-inflammatory biomarkers in macrophages, inhibition of endogenous prostaglandin E2 levels, and reduction of nitric oxide values may alleviate migraine pain (Zargaran et al., 2014 ▶). Chamomile modulates phase I and phase II drug metabolizing enzymes in the liver (Maliakal and Wanwimolruk, 2001 ▶), improves endometrial tissue arrangements (Farideh et al., 2010 ▶), and alleviate pain and edema present in various inflammatory conditions (Tomić et al., 2014 ▶) in animal experiments. The anti-inflammatory effect of chamomile is suggested to be mainly due its essential oils, such as bisabololand, chamazulene and matricin, possibly via inhibition of histamine release and the production of prostaglandin (Safayhi et al., 1994 ▶; Miller et al., 1996 ▶; Srivastava et al., 2010 ▶). 

*Malus orientalis*

Apple (*Malus orientalis*) is advised for treatment of acute general edema, muscle pain, abscess, otitis, purulent wounds, toothache, chronic cough and pneumonia, abdominal pain, and intestinal inflammation as well as for prevention of headache by Avicenna. The apple contains polyphenols with a large variability in their structures, which are stored in vacuoles and chromoplasts (Francini and Sebastiani, 2013 ▶). Antioxidant reactions of phytochemicals inhibit the oxidation of harmful substances and act as radical catcher. Reactive oxygen species are noxious in a large amount and cause cell damage by reaction with lipids, proteins and deoxyribonucleic acid (Mladenka et al., 2010 ▶). Secondary plant metabolites and polyphenols have anti-inflammatory, anti-carcinogenic, anti-microbial, anti-oxidant, and anti-thrombotic effects (Scalbert et al., 2005 ▶; Jelodarian et al., 2013 ▶). Apple polysaccharide extract is suggested to prevent colitis-associated colon cancer via the inhibition of TLR4/MD2-mediated signaling and the inhibition of NF-κB-mediated inflammatory signaling pathways (Zhang et al., 2015 ▶). Apple flavonols in combination with fish oil inhibited the production of pro-inflammatory mediators and significantly improved blood lipid profiles in rats with diet-induced hyperlipidemia and lipopolysaccharide-induced acute inflammation (Sekhon-Loodu et al., 2014 ▶).

 High-flavonoid apple was associated with decreases in the transcription levels of inflammation-linked genes for interleukin-2 receptor, chemokine receptor 2, chemokine ligand 10, and chemokine receptor 10 as well as in production of prostaglandin E2 (Espley et al., 2014 ▶).

*Boswellia serrata*

In medieval Persian, frankincense (*Boswellia serrata*) was advised for treatment of abscess, wounds and malignant tumors, skin rashes, dermatitis, nausea and vomiting, gastrointestinal inflammation, and arthritis. Several experimental studies have shown that frankincense possesses anti-inflammatory, analgesic, antimicrobial, hepatoactive, and anti-proliferative effect (Abdel-Tawab et al., 2011 ▶). 

The resinous part of *Boswellia serrata* possesses several anti-inflammatory substances, including mono-, di-, tri-, tetra-, and four major pentacyclic triterpenic acids (Siddiqui, 2011 ▶). Oral administration of Boswellia serrata gum resin extract significantly reduced the levels of several inflammatory mediators (interleukins 1β and 6, tumor necrosis factor-α, Interferon gamma, and prostaglandin E2), and increased interleukin-10.

 The protective effect of frankincense against rheumatoid arthritis is suggested evident due to the decrease in arthritis scoring and bone histology in a collagen induced arthritis model in rats (Umar et al., 2014 ▶). Aflapin, a novel Boswellia-derived anti-inflammatory product, significantly inhibited interleukins 1β-induced death of human primary chondrocytes and improves production of glycosaminoglycan in human chondrocytes (Sengupta et al., 2011 ▶).

*Cinnamomum camphora*

Camphor (*Cinnamomum camphora*) is advised for treatment of headache and arthralgia as well as against inflammation in different organs. Camphor inhibited heat-sensitive transient receptor potential vanilloid subtype 1 (TRPV1) and several other related channels, which may underlie the analgesic effects of camphor (Xu et al., 2005 ▶). Camphor activated cultured primary keratinocytes (contained heat-activated receptors), and this effect was abolished in TRPV3 null mice (Moqrich et al., 2005 ▶).

Phytochemical investigation of Myrrh (*Commiphera myrrha*) has resulted in identification of more than 300 secondary metabolites which have exhibited a wide-range of pharmacological properties that are effective in treatment of inflammatory and infection diseases. 

The bioactive steroids guggulsterones have been suggested as a potent inhibitory component on tumor cells and inflammation (Shen et al., 2012 ▶). Lavender (*Lavendula stoechas*) is believed to have a variety of therapeutic and curative properties in the Canon. In a placebo-controlled clinical trial, inhalation of lavender oil was suggested as an effective and safe treatment in acute management of migraine attacks (Sasannejad et al., 2012 ▶). Lavender inhibited some inflammatory processes, such as lipopolysaccharide-induced inflammatory reaction (Koulivand et al., 2013 ▶). 

Ethanolic and aqueous extracts of saffron (*Crocus sativus*), another analgesic and anti-inflammatory drug mentioned in the Canon, have been suggested as a useful substances in treatment of different kinds of neuropathic pain and acetaminophen toxicity (Amin and Hosseinzadeh, 2012 ▶; Omidi et al., 2014 ▶.

Several strategies have been used for development of new drugs. One of these strategies is the use, development and improvement of existing medicines, like natural healing substances, which have been used long to treat the illnesses in traditional medicine. Although some of anti-inflammatory and analgesic substances advised by Avicenna in the Canon are used by modern medicine, the exact mechanism of their action as well as biochemical and pharmacological values needs more investigations. Several other drugs are still unexamined, which have the potential for further investigations and discovery of new drugs against inflammatory diseases and pain.
